# Annotations

**Published:** 1896-12-12

**Authors:** 


### Annotations.

### "Ins and Outs."

In spite of all enactments concerning free and compulsory education a certain number of children grow up in almost complete ignorance. The offspring of canal boatmen, of travelling show folks, of tramps and casual paupers, manage to evade the provisions of the Education Act, and the vigilance of School Boards. They are in no one's jurisdiction, and only the efforts of charity when encouraged by the wishes of parents can do anything to dissipate their ignorance. Occasionally they go into the workhouse for a day or two, but soon they can bear its restraints no longer and go off on tramp again, taking their children with them. In a week or two they may be in another workhouse, or even back in the same one. The children thus grow up ignorant and untrained, fit only for the shiftless nomadic lives their parents led before them. Mr. Geoffrey Drage, speaking recently at Whitechapel, dwelt on the condition of these "ins and outs," and expressed the wish that some means could be devised for taking them out of the control of their parents. They should not, however, be shut up in large "barrack" schools, where they come to be regarded, and to regard themselves, only as parts of a great machine, but rather boarded out in some poor but honourable household, where they may learn the ordinary duties and experience some of the affection and discipline of family life.

### Charity and State Aid.

Certain people seem to think that the Government is a source of boundless wealth, and that to throw the hospitals upon the State is to ensure their permanent prosperity. Perhaps if they would look around and consider what an amount of outside pressure it has required to introduce reforms into the Poor Law infirmaries—the existing hospitals of the State—they would hesitate before they recommended the transference of the great charity hospitals of this country to official management. There is an apparent simplicity about the proposal which is very fascinating to those who are not conversant with the difficulties of hospital management, but those who understand these difficulties will rarely be found to look hopefully on such a rough and ready solution of the question. We recently received a letter from Colonel Gordon Watson (which, by the bye, seems to have been at the same time sent to the *Lancet*, where it appeared last week) not only urging that the hospitals should be supported by the State, but allocating a special fund for the purpose, viz., an extra penny on the income tax! But can Colonel Watson really be accepted as an authority on the snbject? To see how far his opinion is worth listening to, we must look at the rest of his proposal, where we find, as we should expect to find, that it is coupled with impossible conditions. "Should this be entertained by the Government all hospitals must be absolutely free, no letter of recommendation necessary, and only the fact that there is no vacant bed should cause a patient to be refused admission." This has a beautiful air of fairness about it which makes it very taking to the ignorant, but every hospital manager must know that under such conditions no general hospital could run for a month. The appalling excess of applications beyond the beds available is one of the difficulties of hospital management which outsiders wot little of, and a free hospital run on the lines suggested by Colonel Watson would be at once filled up with chronics, and be entirely diverted from its proper purpose. Colonel Watson's financial scheme must be coupled with his plan for the management of his State-supported hospitals, and must be judged accordingly. A gentleman writing in one of the evening papers, over the signature "M.B.," equally falls into error, but in the opposite direction. In his State-supported hospitals he would have "the most strict supervision exercised as regards the *bonâ fide* poverty of the patients." But such hospitals we already have. If poverty alone is to be the test, the State already possesses hospitals under the Poor Law. Charity, however, has declared that hospitals are wanted for people who do not come under such strict limitations as to poverty, and we feel assured that if all the hospitals were taken over by the State to-morrow, and governed by the iron rule of officialism, charity would step in again and build hospitals afresh, unless the doing so were rendered penal.

### Bacteriology and Agriculture.

The non-scientific Briton, even of the educated class, has a certain amount of hardly-disguised contempt for the bacillus and all who are in any way connected with him. And yet there is not a man of science anywhere who would venture to set bounds to the possibilities of disease prevention opened out by the science of bacteriology. It is not man alone who will be advantaged by the progress of bacillary knowledge. Animals also are likely to benefit. We are glad, therefore, on the one hand, that the German Government is taking steps to give the animal and agricultural worlds the advantages of our present knowledge of things bacteriological, and exceedingly sorry, on the other, that our own Government, like Gallio, "cares for none of these things." Dr. Koch is under commission from the German Government to inquire into the circumstances of the cattle plague which is now raging in South Africa, and, if possible, to devise some means of prevention. But what are we doing? The Foreign Office, it is stated, was warned long ago, before the plague crossed the Zambesi, of its probable deadly effect; and of the possibility of checking its onward march. But no notice whatever was taken of the warning. Now, science is really destined to be the guiding force of the future in all questions of practical moment; and a Government which does not recognise this is serving the interests of its country very badly. When it is remembered what a very small periodical outlay, probably a very few thousand pounds annually, spent in paying specialists and furnishing them with facilities for research, would give agriculture and other important arts the unspeakable advantage of every bit of knowledge of a practical kind to which science has attained, it is impossible to feel anything but profound surprise that the few thousands a year are not so spent. It is a great pity that there are not more men of science in the House of Commons. It is more than shameful that the general progress of one of the most important industries in the country should be endangered for want of a little special knowledge, knowledge which could be easily obtained by a moderate expenditure.

